# Eating disorder risks and awareness among female elite cyclists: an anonymous survey

**DOI:** 10.1186/s13102-022-00563-6

**Published:** 2022-09-23

**Authors:** C Koppenburg, F Saxer, W Vach, D Lüchtenberg, A Goesele

**Affiliations:** 1 Department of Sport Sciences, University of Konstanz, Postfach 30, D-78457 Konstanz, Germany; 2 Crossklinik Basel, Bundesstr. 1, CH-4054 Basel, Switzerland; 3 Basel Academy for Quality and Research in Medicine, Steinenring 6, CH- 4051 Basel, Switzerland; 4 University of Basel, Petersplatz 1, CH-4001 Basel, Switzerland; 5 Novartis Institutes for Biomedical Research, Novartis Campus, CH-4051 Basel, Switzerland

**Keywords:** Female, Elite cyclists, Eating disorder, EAT-26, Awareness, Anonymous survey, Ideal weight

## Abstract

**Background:**

Eating disorders (EDs) are an increasingly recognized concern in professional sports. Previous studies suggests that both female gender and endurance sports put athletes at risk. Female elite cyclists are hence of specific interest. The present study aimed at evaluating the distribution of the individual risk of ED in this group. Further the association between individual risk and both the awareness for the topic ED and the deviation from “normal” weight was depicted.

**Methods:**

Female cyclists registered with the Union Cycliste Internationale were contacted via email or facebook and asked to complete a survey comprising age, weight, the Eating-Attitude-Test (EAT-26), and questions regarding ED awareness. The observed distribution of the EAT-26 score was compared to results from previous studies in normal subjects and athletes. The distribution of the ED awareness was described and ED awareness was correlated with the EAT-26 score. Both the deviation from ideal weight and the body mass index (BMI) were correlated with the EAT-26 score.

**Results:**

Of the 409 registered athletes 386 could be contacted, 122 completed the questionnaire. Age ranged from 20-44yrs, BMI from 17.0 to 24.6 kg/m^2^. In the EAT-26, 39 cyclists (32.0%) scored above 20 points indicating a potential benefit from clinical evaluation, 34 cyclists (27.9%) scored 10–19 points suggesting disordered eating. Sixteen athletes (13.2%) had been treated for an ED. About 70% of athletes had been pressured to lose weight. The mean EAT-26 score was above the average observed in normal female populations. It was also above the average observed in many female athlete populations, but lower than in other leanness focussed sports. More than 80%of athletes perceived elite cyclists at risk for developing ED. Increased ED awareness and deviation from the ideal weight were associated with higher EAT-26 scores, but not the body mass index.

**Conclusion:**

Female cyclists are at risk of developing ED and they are aware of this risk. To improve their health and well-being, increased efforts to support elite cyclists and their teams in preventive activities and early detection are crucial.

**Supplementary Information:**

The online version contains supplementary material available at 10.1186/s13102-022-00563-6.

## Introduction

Body-shape and body (dis)satisfaction are relevant problems in society with many women regularly dieting [1–3]. Food behaviour in athletes has also gained increasing awareness [4–6]. There are concerns about the number of reports on eating disorders (ED) and disordered eating (DE) among elite but also recreational athletes [7, 8], coinciding with a growing preoccupation with nutrition and fitness in the media and general population [9–11]. The term “eating disorder” refers here to diagnoses such as anorexia nervosa or bulimia nervosa, which are defined based on specific, narrow criteria. In contrast, the term “disordered eating” describes a range of irregular eating behaviours, that may or may not allow to diagnose a specific eating disorder [12].

In the context of DE and ED the literature differentiates between predisposing factors, trigger factors and perpetuating factors [13]. Biological factors such as age, pubertal status, genetics, psychological profile (perfectionism, self-esteem, negative affect etc.) and socio-cultural aspects (body ideal, peer pressure or family influence etc.) are regarded as predisposing factors [4, 14, 15].Trigger factors can be pubertal changes that make it difficult to achieve perceived weight or body-shape requirements [16]. External comments (e.g. by coaching staff), injuries leading to reduced training intensity or weight class requirements are additional examples [16–18]. Finally, positive feedback after weight-loss, or the initial improvement in performance play important roles as perpetuating factors [4]. To efficiently prevent the development of DE or ED at the individual but also at the system level of professional sports, awareness for the topic is paramount.

The risk of transgressing from normal via suspicious eating behaviour (or DE) to a manifest ED is multifaceted. Among the specific factors often discussed in the literature, gender seems the one most analysed. Females are reported to be affected more often than males both in the general population (5–10%) and among athletes (up to 42%) [5, 19, 20]. Underreporting in males is thought probable for both the athlete and the general populations [21, 22]. However, when focusing on dieting behaviour alone, Prnjak et al. [6] could not find a difference between female and male competing athletes. Another potentially important factor may be the type of sport. Most authors report higher rates of DE/ED in endurance, aesthetic sports and sports with weight classes [16], while others could not establish respective correlations and focussed more on factors such as body dissatisfaction and perfectionism [6, 8]. Perfectionism actually is a multifaceted personality trait that is relatively prevalent in successful athletes, however also often observed in the context of DE/ED [23, 24]. There is a fine line between adaptive (healthy) and maladaptive (unhealthy) perfectionism. Both seem to increase female athletes’ vulnerability for body dissatisfaction, while, in men the association between these personality traits could not be established [6].

To provide a basis for the successful prevention, diagnosis, and treatment of ED in elite athletes, epidemiological studies are important. They allow the investigation of the distribution of the individual risk and the identification of potential risk factors. Corresponding studies have been conducted for many specific sports. For example, DeBate et al. [25] investigated triathletes, Bachner-Melman et al. [26] aesthetic athletes, Schterscherbyna et al. [27] elite swimmers, Wyon et al. [28] elite student and professional ballet dancers, Prather et al. [29] female soccer players, Neves et al. [30] artistic gymnasts, and Michael et al. [31] recreational and competitive rock climbers. These studies provide an excellent basis for a comparison with further sports.

The first aim of the study was to provide a description of the distribution of the individual ED risk among female elite cyclists. Similar to other sports, there is some focus on leanness in this group. However, there is still room for a variation in body weight and body stature – also depending on the specialization. This may increase the risk that an individual DE or ED remains undetected. Furthermore, there is an additional, simultaneous focus on endurance and muscle power, which may neutralize the focus on leanness. Hence there is an interest in a comparison with other sports, which was also a topic of this study. The second aim of the study was to investigate the distribution of the individual degree of awareness of the athletes for the topic of EDs in this group and its potential association with the individual ED risk. The third aim was to investigate the association of the deviation from “normal” body weight with the individual ED risk.

## Methods

### Study design

Female cyclists registered with the Union Cycliste Internationale (UCI) were identified via the web (http://www.uci.ch/road/teams accessed January 9th 2018). Of the 409 registered athletes 368 (90.0%) could be contacted via email or facebook. Using the same contact mode, they were sent a link for an online survey (www.onlineumfrage.com), asking them to complete the questionnaire anonymously. Anonymity was ensured since relevant response bias has been reported in open surveys [32, 33].

### Design of the questionnaire

The questionnaire used in the survey is documented as Additional File 1.

The survey started with questions on year of birth, height, current weight, and personal ideal weight. Then, the 26 items of the EAT-26 (Eating Attitude Test [34]) were presented with a neutral introduction. Next, four items on DE behaviour in the last six months were listed with responses on a 6-point Likert scale, followed by binary questions about a weight loss of more than 8 kg in the last 6 months, previous treatment for ED, the eleven features of anorexia athletica [35, 36] and five questions about the awareness for the topic EDs.

The four items on DE behaviour reflect four aspects widely discussed in the literature: binge eating, self-induced vomiting, abuse of laxatives or diuretics, and overtraining [5, 37, 38]. The five questions on the awareness were chosen to get an impression of the perception of the topic ED among the athletes. They were self-designed, as we were not aware of previous studies addressing this issue.

For each athlete the date of completion was recorded, allowing the determination of age. The duration of time taken to complete the questionnaire was also documented. The body mass index (BMI) was calculated based on self-reported height and weight.

### Score definitions

The psychometric properties of the EAT-26 have been evaluated [34] and the instrument has been validated against other instruments [39–41]. This holds for both the general population and athletes [33]. It has been used in many studies investigating the DE/ED risk in athletes. All 26 items allow the response categories: ”never”, “rarely”, “sometimes”, “often”, “usually” and “always”. The first three are scored as 0 and the following as 1, 2, and 3 respectively. Subjects with a score of 20 or above should be further investigated for diagnostic criteria of EDs [41, 42]. Scores between 10 and 19 points might be suggestive of DE. Additionally, EAT-26 responses generate three sub-scores reflecting the constructs “dieting”, “bulimia and food preoccupation” and “oral control”. According to Garner et al. [34] the first construct relates to “an avoidance of fattening foods and a preoccupation with being thinner”, the second covers “items reflecting thoughts about food as well as those indicating bulimia”, and the third relates to “self-control of eating and the perceived pressure from others to gain weight”.

The results of EAT-26 and similar instruments have to be interpreted with caution as they represent screening tools rather than diagnostic criteria. Consequently, in the context of this study the EAT-26 is used as a standardised self-reported questionnaire and the values are interpreted as measurements of the individual ED risk.

To summarize the information from the five awareness items, a simple summary score was defined ad hoc. For each “yes” answer one point was assigned. However, the questions “Do you know people with eating disorders?” and “If so, are those female cyclists?” were evaluated together and a point was given only if both questions were answered with “yes”. The resulting summary score is referred to as “ED awareness score” and can range from 0 to 4.

### Analytical strategy

We start with describing basic characteristics of the study population. In order to describe the distribution of the individual ED-risk, the distribution of the individual EAT-26 score values in the study population is considered and contrasted to results from other populations. First the quantiles of the overall score and the three sub-scores are compared with population norms and corresponding results from anorexia nervosa (AN) patients published by Garner et al. [34]. Second, the mean and standard deviation of the overall score is compared with a series of publications using EAT-26 in different athlete-populations. Finally, the distribution of the DE behaviour items is described.

Next the distributions of the awareness items are depicted and a potential relation between unease in filling out the questionnaire and the time used to complete the questionnaire is investigated. To address the association between awareness and individual ED risk, the correlation between the ED awareness score and the EAT-26 summary score is investigated. Finally, the association between deviation from “normal” weight and the individual risk of ED is analysed. Two candidate variables are considered to express the deviation: The BMI and the deviation from the personal ideal weight. The association is depicted by the correlation with the EAT-26 summary score. The deviation from the personal ideal weight is defined as the ratio between the actual weight and the reported personal ideal weight.

### Statistical methods

The distribution of continuous variables is described by histograms. The distribution of ordinal variables is described by stacked bar charts. The distribution of binary variables is described by bar charts. The association between variables is illustrated by scatter plots and is quantified by the Pearson correlation coefficient. For the Pearson correlation the p-value of testing the null hypothesis of no association is reported. This p-value is based on the Wald-test principle assuming a t-distribution for the test-statistic. The degree of association visible in a scatter plot is further illustrated by a regression line.

All computations are performed with Stata 15.1.

## Results

### Participants and treatment for ED

Among the 386 athletes contacted, 136 (37.0%) started the questionnaire, however 14 stopped after the initial questions. The remaining 122 athletes (89.7%) responded to all items of the EAT-26 and form the population for this analysis. One athlete stopped after completing the EAT-26, and another stopped after responding to the DE behaviour questions. The remaining 120 athletes responded to all items. The median duration for completion of the questionnaire was 7 min. The questionnaires were answered in the time period between March 19, 2018, and April 20, 2018.

The participants were between 20 and 44 years old with an average of 25.2 years (SD 4.5 years). The BMI ranged from 17.0 to 24.6 kg/m^2^. Twelve (10.0%) of the participants had a BMI below 18.5, and further 26 (22.5%) had a BMI lower than 19.5. Sixteen athletes (13.2%) reported having been treated for an ED.

### EAT-26

**Fig. 1:**
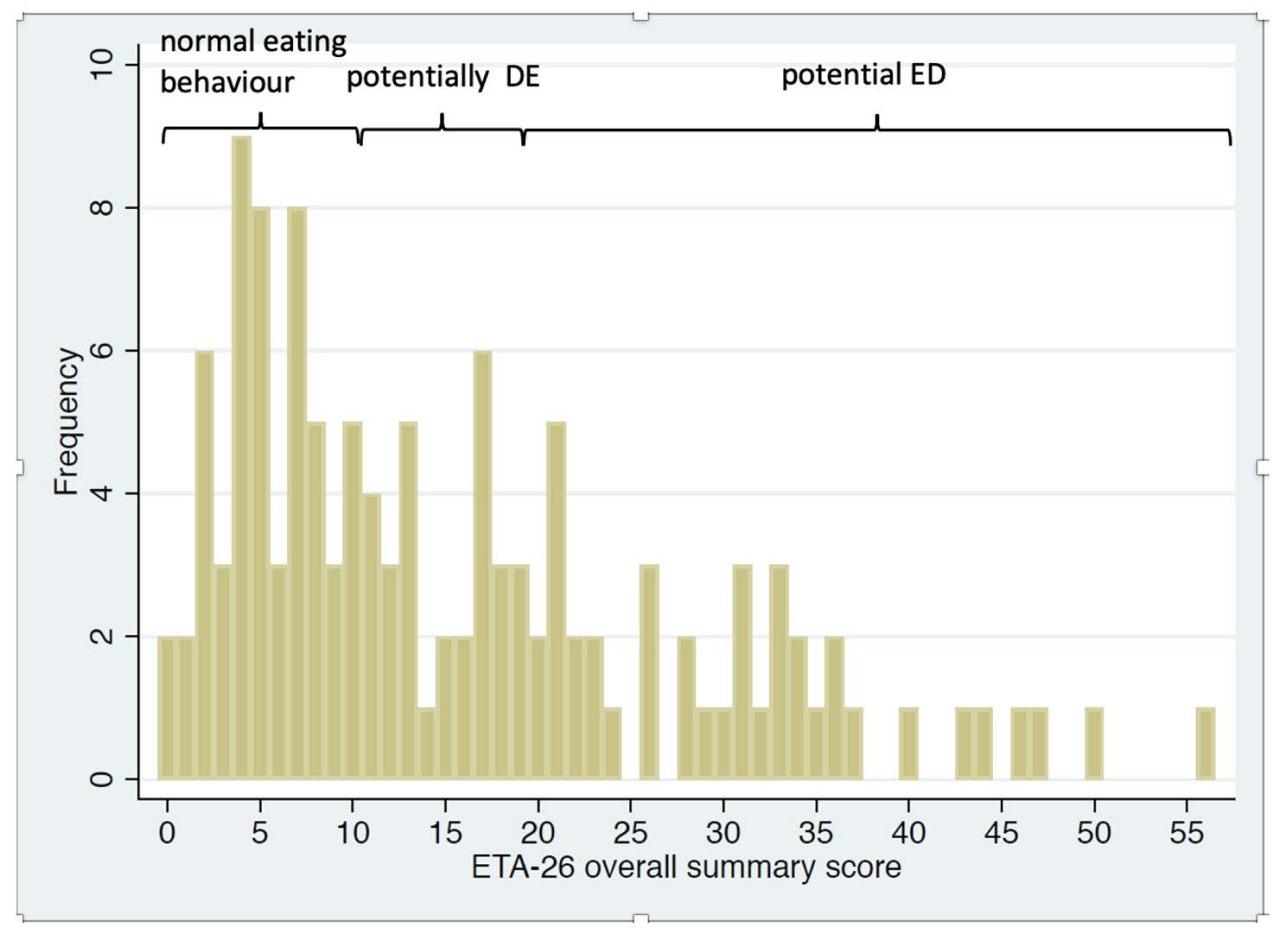
The distribution of the individual EAT-26 overall score values in the study population

The distribution of the EAT-26 summary score is shown in Fig. 1. In total, 39 athletes (32.0%) scored 20 or above which renders them eligible for further diagnostic evaluation. In addition, 34 athletes (27.9%) scored between 10 and 19. Seven athletes (5.7%) scored particularly high values between 40 and 60.

**Fig. 2:**
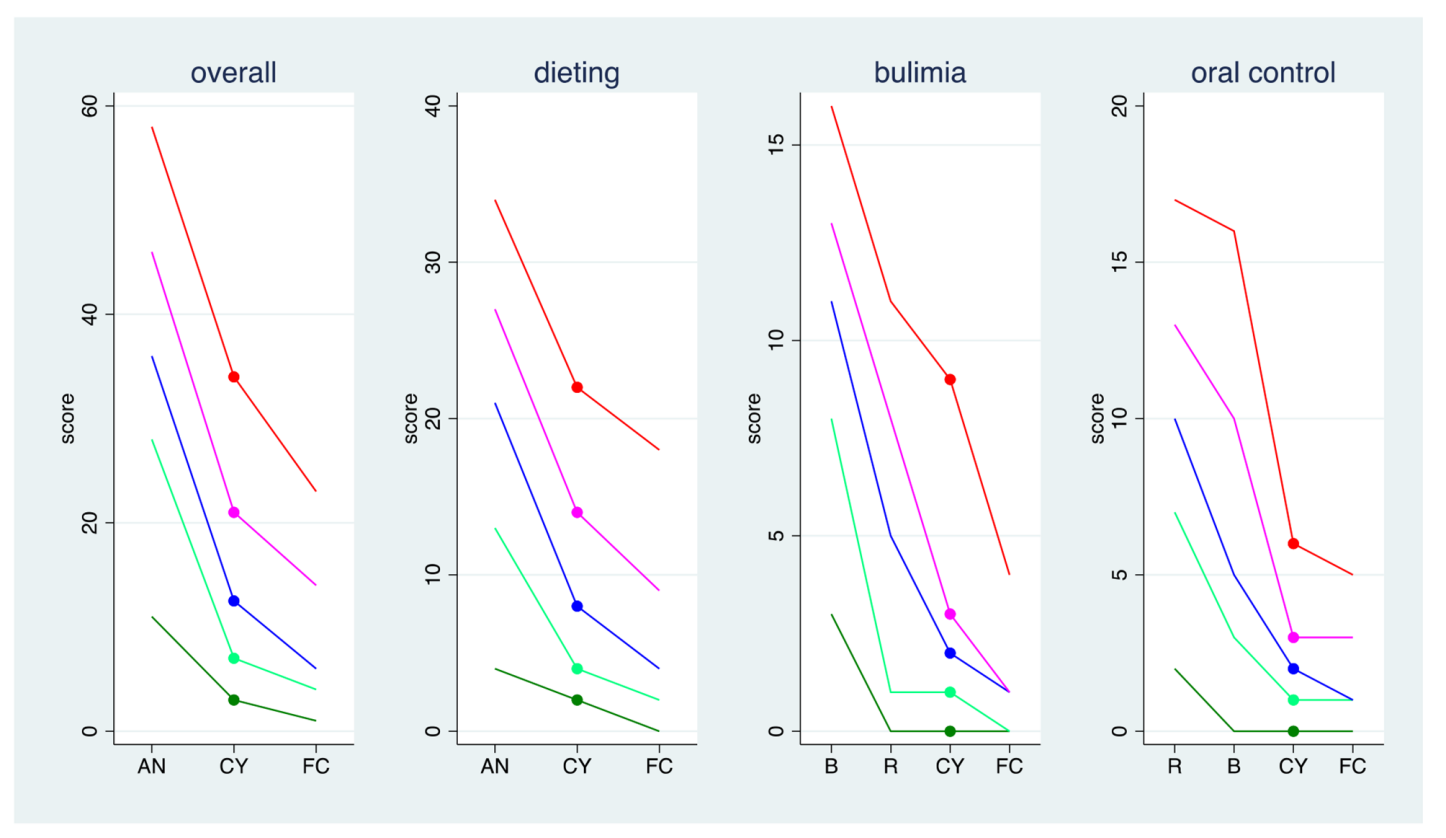
Comparison of EAT-26 sub scores of the present sample of cyclists with normal and pathologic values based on previous research (n = 120). Comparison of the 90th (red), 70th (purple), 50th (blue), 30th (light green) and 10th (dark green) percentiles in the study population (CY) with a female control (FC) population and a population of anorexia nervosa (AN) patients. For two subscores, the AN population is divided into bulimic AN patients (B) and restrictive AN patients (R). The numbers shown for the comparison groups are based on the publication by Garner et al. [34]

The distribution of the overall score and the three sub-scores is shown in Fig. 2. This figure also presents a comparison to the distributions in a normal control group and a group of anorexia nervosa (AN) patients previously studied by Garner et al. [34]. For all scores, we observed that our study population tended to score higher than the normal control group but lower than AN patients. There are, however, qualitative differences. With respect to oral control, our study population is close to the normal population and differs distinctly from AN patients, in particular from restrictive AN patients. For the overall score and the dieting score, the study population was closer to normal controls than to AN patients. With respect to the bulimia score, the study population was close to the average between the restrictive AN patients and the normal controls, but was still scoring distinctly lower than bulimic AN patients.

**Fig. 3:**
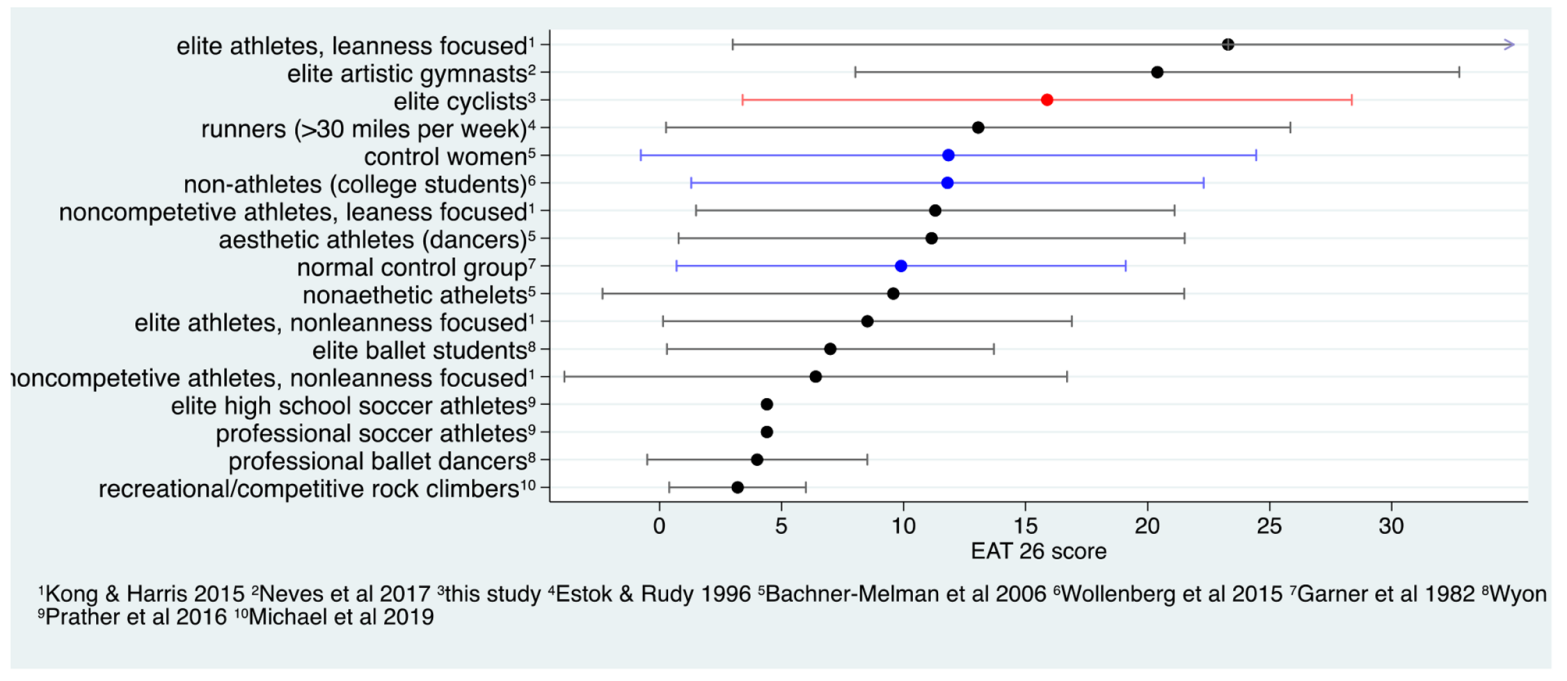
Comparison of EAT-26 scores from various populations. Mean (+/- SD) of the EAT-26 summary score in various populations of female athletes and controls. This study is indicated in red. Control groups are indicated in blue

A comparison of our study population with other populations of female athletes/controls is pictured in Fig. 3. Female athlete groups such as rock climbers, professional ballet dancers, elite ballet students or soccer players tended to score lower than the normal population in the EAT-26. Cyclists tended to score on average higher than the normal population, but still lower than athletes performing artistic gymnastics. Compared to groups of athletes from various disciplines focusing on leanness, female cyclists scored lower than elite athletes and higher than non-competitive athletes.

### DE behaviour

**Fig. 4:**
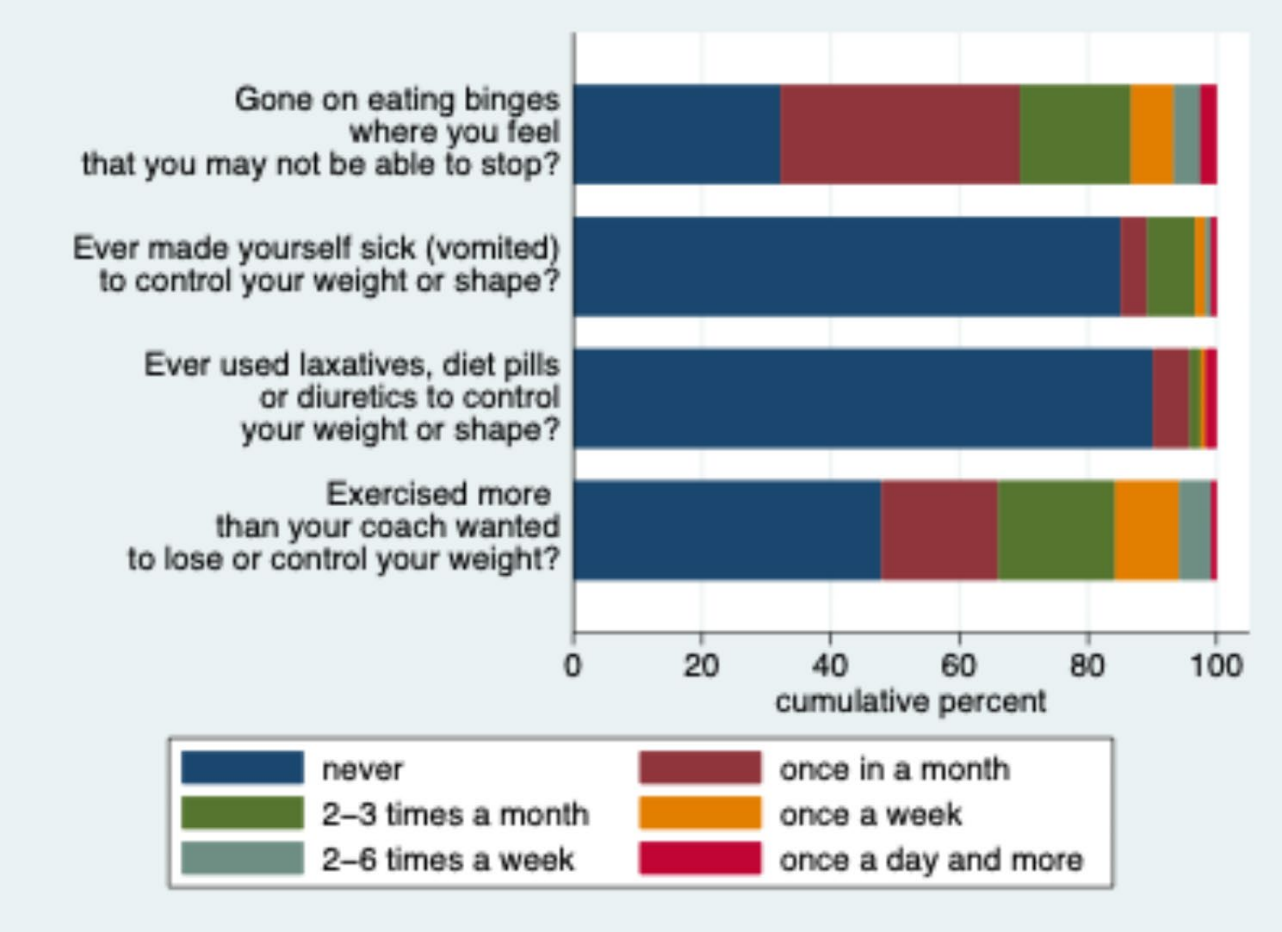
Distribution of responses to four items about DE behaviour in the study population (n = 121). The numerical frequencies behind this graph are available in Additional File 2

DE behaviour was observed in more than half of all athletes, especially with respect to eating binges and overtraining (Fig. 4). However, these behaviours were typically reported to happen at a low rate with less than ¼ of athletes reporting these tendencies once per week or more often. Also attempts to stimulate vomiting or the use of medication were rather rare.

Furthermore, 6 (5.0%) athletes reported a weight loss above 8 kg within the past 6 months.

### Awareness

**Fig. 5:**
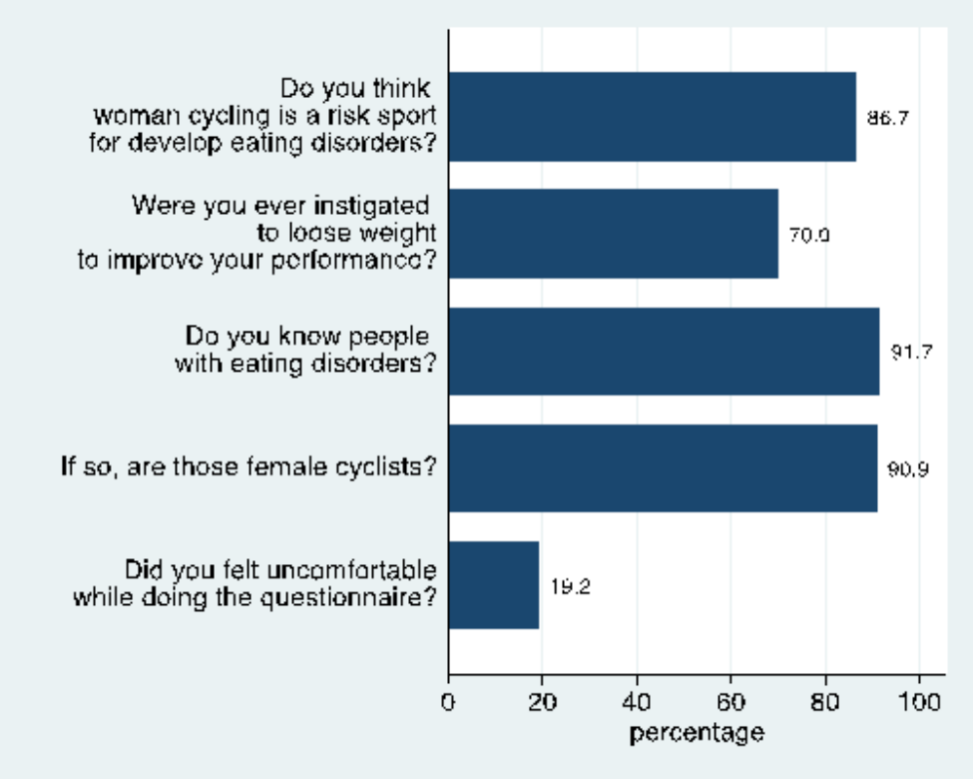
Frequency of answering with “yes” for five questions about ED awareness in the study population (n = 120)

According to Fig. 5 more than 80% of the athletes considered cycling a high-risk sport for developing DE/ED and about 90% of participants knew persons with ED, typically fellow female cyclists. More than two thirds of athletes reported that they had been told to lose weight in order to improve their performance. Finally, about 20% admitted discomfort while completing the questionnaire.

Among the 23 athletes who admitted feeling uncomfortable, 8 (34.8%) filled the questionnaire within five minutes and 7 (30.4%) needed more than ten minutes. The corresponding percentages in the remaining athletes were 19.6% and 20.6%, indicating that an unease may result in a specifically quick or deliberate completion of the questionnaire.

**Fig. 6:**
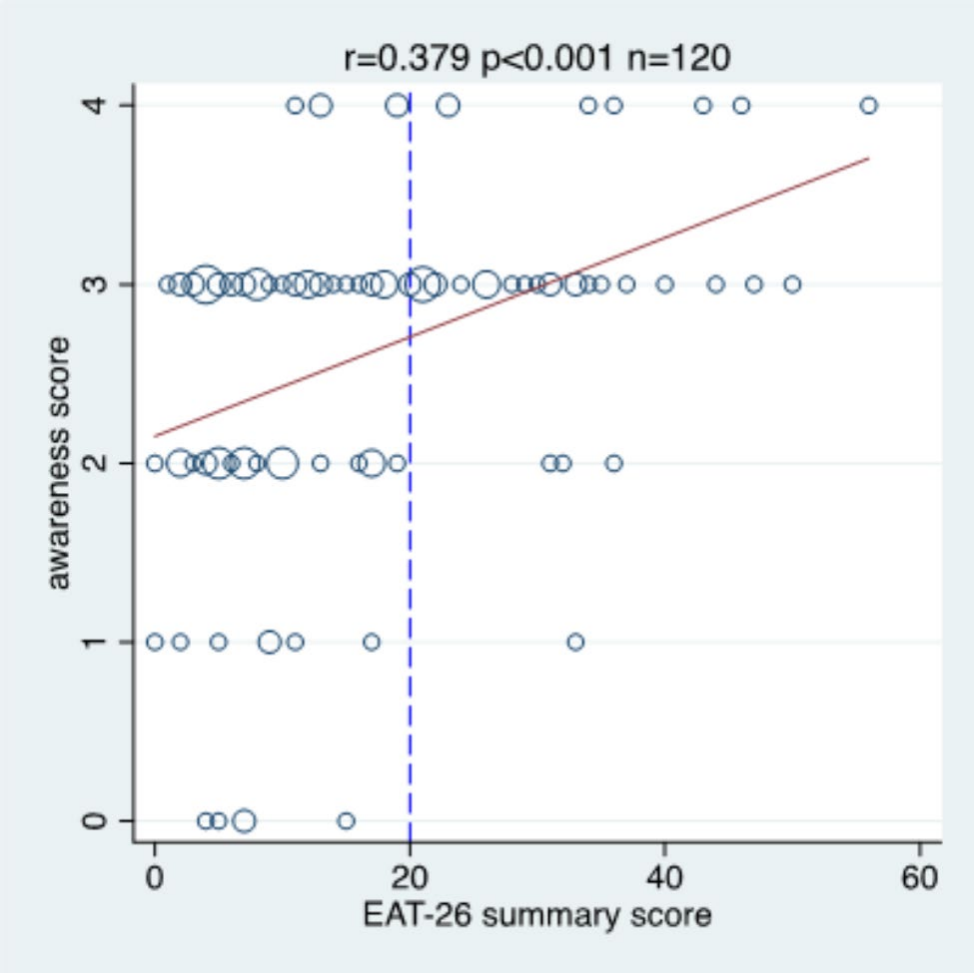
The association between the EAT-26 summary score and the awareness score. r = Pearson correlation coefficient; p = p-value of testing the null hypothesis of no correlation; n = sample size; the **red line** indicates the regression line; the **dashed blue line** marks the level 20 for the EAT-26 summary score.

Figure 6 depicts the association between the ED awareness score and the EAT-26 score. It indicates a moderate association (r = 0.379), and 89.5% of the athletes with an overall EAT-26 score of at least 20 scored at least 3 points on the awareness score.

### Association of weight deviation with the risk of ED

Five (4.1%) participants reported an actual weight below their personal ideal weight, in 14 respondents (11.5%) the actual and ideal weights matched. When considering the deviation defined as the ratio between the actual and the ideal weight, 66 participants (54.1%) showed a ratio between 1.0 and 1.05, 30 (24.6%) a ratio between 1.05 and 1.1 and 7 (5.8%) a ratio above 1.1.

**Fig. 7:**
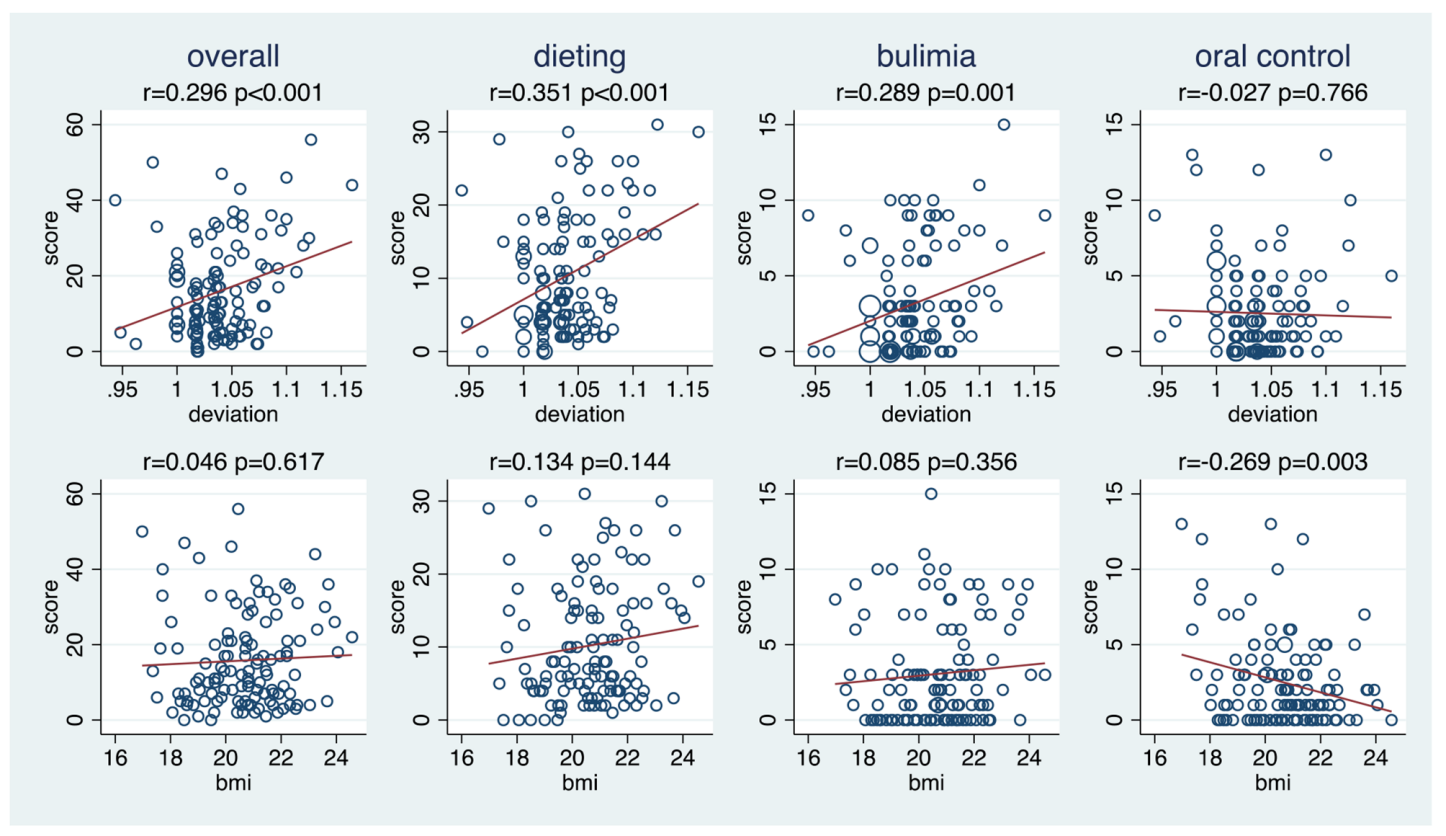
The association of the EAT-26 overall score and the three sub-scores with the deviation ratio and with the body mass index (n = 120 for BMI, n = 121 for deviation). r = Pearson correlation coefficient; p = p-value of testing the null hypothesis of no correlation. BMI = body mass index

**Fig. 8:**
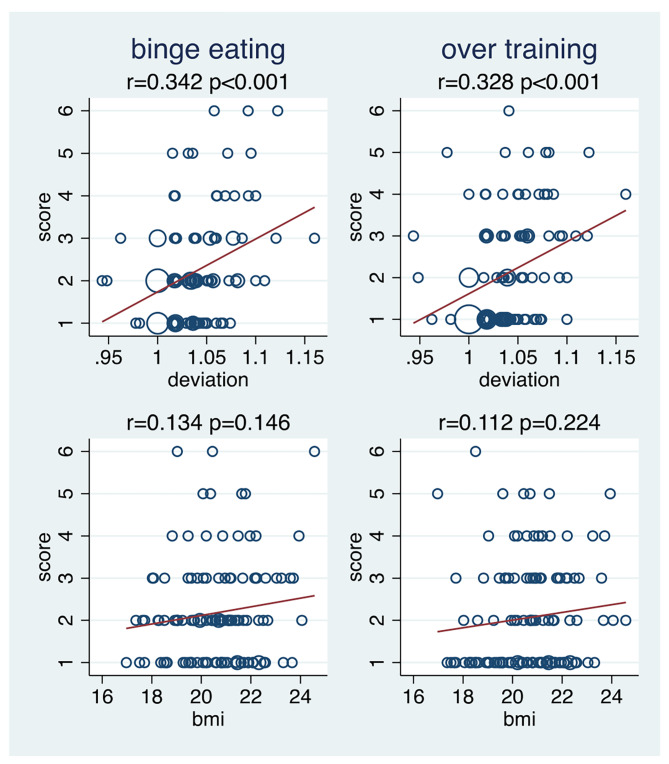
The association of binge eating and overtraining with the deviation ratio and with the body mass index (n = 120 for BMIi, n = 121 for deviation). r = Pearson correlation coefficient; p = p-value of testing the null hypothesis of no correlation. BMI = body mass index

Figure 7 depicts the relationship of the deviation-ratio and the BMI to the EAT-26 overall score and the three sub-scores. The deviation-ratio is distinctly associated to the overall score, the dieting and bulimia sub-scores, but not to the oral control score. In contrast, BMI is only very weakly associated to the first three scores and distinctly negatively associated with the oral control score. The much more pronounced association with the deviation-ratio than with the BMI could be also observed for the two DE behaviour items with a substantial variation in the study population (Fig. 8).

## Discussion

First, this study aimed at providing a description of the distribution of the individual ED risk among female elite cyclists and to compare it with the distribution in other populations. Second, the study aimed at investigating the distribution of the individual degree of awareness of the athletes for the topic of ED and its potential association with the individual ED risk. Third, the study aimed at investigating the association of the deviation from “normal” body weight with the individual ED risk.

### Individual risk of ED

This study confirms that female elite cyclists are at risk of developing ED. 13% of the participants reported to have been treated for ED, and nearly one third scored 20 or more on the EAT-26 overall score. More than half of the participants showed at least suspicious EAT-26 scores and reported binge eating or overtraining at least once per month. The EAT-26 scores were distinctly higher than in normal controls, although far from those observed in AN patients. These data imply that DE/ED constitutes an important and common problem within the population of female cyclists.

The comparisons with published results conducted as part of this study clearly indicate an increased risk of DE/ED in elite female cyclists compared to the general female population of comparable age. This is in contrast to other elite female athletes who can be at a lower risk than the general population – depending on their sport. Elite artistic gymnasts and leanness focused elite athletes were the only female athletes found to be at higher risk than cyclists. These results are in line with previous studies investigating the effect of sport type on the development of DE/ED. In particular, Sundgot-Borgen [35] analysed the frequency of clinically confirmed ED in female elite athletes among six sport groups and found the highest rates of ED in aesthetic sports and weight dependent sports followed by endurance sports (including cycling). Our study allowed to contrast results in female cyclists with published results from elite artistic gymnasts, indicating that the additional focus on endurance and muscle power may reduce the risk of ED.

However, a comparison of mean values of the EAT-26 score does not show the full picture, as the population distributions are typically highly skewed. Therefore, the comparison of the frequency of athletes with an EAT-26 score of 20 or above can add additional insights. Kong & Harris [43] reported a frequency of 49% in 80 elite leanness-focused female athletes and of 8% in 48 elite non-leanness-focused female athletes. Schtscherbyna et al. [27] reported a frequency of 7.7% in 78 elite female swimmers, Wollenberg et al. [44] a frequency of 6.6% in athletes and of 16.5% in non-athletes in female college students. Prather et al. [29] identified only 1 subject with an EAT-26 ≥ 20 in 220 female elite soccer players. These results indicate, that in the group of female elite cyclists, the frequency of persons at risk for developing DE/ED who might benefit from a referral to clinical specialist evaluation is higher than in other sports.

### Awareness

The results of this study indicate that most female elite cyclists are aware of the risk of ED associated with their sport. More of 70% of the women reported to have been told to lose weight, which can present a relevant trigger factor for the development of DE/ED [16–18]. Nearly all participants were aware of fellow female cyclists suffering from ED. One in five participants also admitted to having felt uncomfortable when completing the questionnaire.

It is interesting to note that an increased risk of ED according to the EAT-26 was clearly associated with an increased awareness, implying that those at risk are aware of the potential problem.

### Relationship between the risk of ED and deviation from “normal” weight

A distinctly increased risk of suspicious EAT-26 scores was observed with increasing deviation of the actual weight from the personal ideal weight, whereas the BMI showed nearly no association. The BMI was negatively associated with oral control, suggesting that loss of oral control is associated with higher weight. In this respect it appears that elite female cyclists seem to be similar to the general population [45].

The association between the deviation from the personal ideal weight and the risk of ED is in line with research pointing to the relevant role of body satisfaction in the development of DE/ED [6]. It is also well aligned to the central role of items corresponding to body satisfaction in screening tools, for example those of the Preparticipation Physical Evaluation Monograph [46], of the Female Athlete Triad Coalition [47] and within the Intermountain Healthcare Management of Eating Disorder Care Process Model [48].

### Limitations

Not all elite cyclists registered with the UCI could be contacted, and only. about one third of contacted athletes participated in the survey. Consequently, the sample may not be representative of all female elite cyclists. We can only speculate about the direction of the potential bias. Athletes with a high risk for ED may hesitate to participate in a survey where they are asked to reflect on their situation and to admit their personal problems. On the other hand, the anonymity of the survey may encourage participation by high-risk athletes, as they can share their problem without the fear of negative consequences. Possibly, these two effects have counterbalanced each other.

The assessment of the BMI and of the deviation from the ideal weight is based on self-reported body weight (and height), and intentional misreporting of the body weight cannot be excluded. In interpreting the results with respect to the BMI, it is hence essential to remember that they refer to self-reported BMI. The deviation from the ideal weight is probably less affected by such a misreporting, as the question about the ideal weight followed the question about the current weight.

The distributions and associations described may vary in dependence on the time on sports. Corresponding information has not been collected as part of this study. Therefore, it was not possible to perform corresponding stratified analyses.

The anonymity may have been beneficial in reducing the risk of selection and reporting bias, however, it renders further investigations impossible and in particular prevents the invitation of participants to a clinical assessment. The need of such an assessments following self-reported screening to arrive at firm conclusions about the prevalence has been emphasized in the literature ([4, 5, 19, 49, 50]).

### Implications

Prevention, early diagnosis and treatment of ED have been identified as important steps towards improving the health and well-being of elite athletes, which has also been the focus of several political statements [51–53]. However, until now this does not seem to have had a broad practical impact on the community of elite female cyclists, as many athletes still appear to be at risk of developing ED and most cycling teams do not have professional staff counselling athletes on nutritional aspects of their sport.

This is surprising to a certain extent, as our investigation reveals a high awareness for the topic ED among female elite cyclists, especially in those at risk. Moreover, there are simple indicators of increased risk such as deviation from the personal ideal weight or certain eating behaviours. In principle, these are favourable pre-conditions for the implementation of prevention and screening interventions. Nevertheless, there still seems to be a great need to improve ED prevention, detection and treatment activities in this group, which should of course include the whole team around each cyclist [54].

## Conclusion

Female elite cyclists are at risk to develop DE/ED. They are aware of this risk and there are simple indicators allowing identification of athletes at risk. To reduce the risk, increased efforts are needed to support elite cyclists and their surrounding teams in prevention activities and early detection.

## Electronic supplementary material

Below is the link to the electronic supplementary material.

Additional File 1: The items of the anonymous survey. This pdf-file presents the items used in the anonymous survey.

Additional File 2: Distribution of the four items on disordered eating behaviour. This pdf-file presents the distribution of the four items on disordered eating behaviour in numerical form.

## Data Availability

The data set used during the current study is available from the corresponding author on reasonable request.

## Declarations

### Author’s information

Clara Koppenburg is a professional rider of the Cervelo Bigla Pro Cycling team. The study was part of her bachelor thesis prepared at the Department of Sport Sciences at the University of Konstanz. Franziska Saxer is a trained general, trauma and orthopaedic surgeon and currently works in osteoarthritis research. She is member of the Medical Faculty of the University of Basel and member of the Basel Academy for Quality and Research in Medicine. Werner Vach is educated in biostatistics and clinical epidemiology. He is currently Senior Researcher in Applied Methodology at the Basel Academy for Quality and Research. Dietmar Lüchtenberg is lecturer and member of the scientific staff in Training and Coaching at the Department of Sport Sciences at the University of Konstanz and has supervised the bachelor thesis. Andreas Goesele is CMO at the Swiss Olympic Medical Center, crossklinik Basel. He was Swiss Olympic Medical Team Doctor at various Olympic games.

## Abbreviations

AN: Anorexia nervosa.
BMI: Body Mass Index.
DE: Disordered Eating.
ED: Eating Disorder.
UCI: Union Cycliste Internationale.
