# Supplementary material for: Eating disorder risks and awareness among female elite cyclists: an anonymous survey
Source: BMC Sports Sci Med Rehabil. 2022 Sep 23;14:172. doi: 10.1186/s13102-022-00563-6 (PMC9502946; doi:10.1186/s13102-022-00563-6)
Supplement: Supplementary file 1 — Additional File 1: The items of the anonymous survey. This pdf-file presents the items used in the anonymous survey. [file 13102_2022_563_MOESM1_ESM.pdf]

Additional file 1 regarding manuscript

Eating disorder risks and awareness among female elite cyclists: an anonymous survey

The items of the anonymous survey

1. Year of birth

2. Height in cm

3. Weight in kg

4. My personal ideal weight in kg

5. Please check a response for each of the following statements (Response options always, usually, often, sometimes, rarely, never)

1. I am terrified about being overweight.

2. I avoid eating when I`m hungry.

3. I find myself preoccupied with food.

4. I have gone eating binges where I feel that I may not be able to stop.

5. I cut my food into small pieces.

6. I aware of the calories content of foods that I eat.

7. I particularly avoid food with a high carbohydrate content.

8. I feel that others would prefer if I ate more.

9. I vomit after I have eaten.

10. I feel extremely guilty after eating.

11. I am occupied with a desire to be thinner.

12. I think about burning up calories when I exercise.

13. Other people think I´m too thin.

14. I am preoccupied with the thought of having fat on my body.

15. I take longer than others to eat my meals.

16. I avoid foods with sugar in them.

17. I eat diet foods.

18. I feel that food controls my life.

19. I display self-control around food.

20. I feel that others pressure me to eat.

21. I give too much time and thought to food.

22. I feel uncomfortable after eating sweets.

23. I engage in dieting behavior.

24. I like my stomach to be empty.

25. I have the impulse to vomit after meals.

26. I enjoy trying new rich foods.

6. In the past 6 months have you: (Response options never, once a month or less, 2-3 times a month, once a week, 2-6 times a week, once a day or more)

*1. Gone on eating binges where you feel that you may not be able to stop?**

2. Ever made yourself sick (vomited) to control your weight or shape?

3. Ever used laxatives, diet pills or diuretics (water pills) to control your weight or shape?

4. Exercised more than 60 minutes a day to lose or to control your weight?

*Defined as eating much more than most people would under the same circumstances and feeling that eating is out of control.

7. Did you lost 20 pounds (9-10kg) or more? (Response options yes/no)

8.. Have you ever been treated for an eating disorder? (Response options yes/no)

9. The Criteria for Anorexia Athletica (Response options yes/no)

1. Limiting intake of "bad" foods for weight loss.

*2. Delayed puberty**

3. Menstrual dysfunction

4. Gastrointestinal complaints

5. Absence of medical illness or affective disorder explaining the weight reduction

6. Distorted body image

7. Dieting to improve performance

8. Restriction of energy intake (energy deficit after training)

9. Bingeing (weight loss and suddenly weight gain)

10. Compulsive exercise

10. General questions (Response options yes/no)

1. Do you think woman cycling is a risk sport for develop eating disorders?

2. Where you ever instigated to lose weight to improve your performance?

4. Do you know people with eating disorders?

5. If so (YES in question 4), are those female cyclists?

6. Did you felt uncomfortable while doing the questionnaire?
