# Supplementary material for: Eating disorder risks and awareness among female elite cyclists: an anonymous survey
Source: BMC Sports Sci Med Rehabil. 2022 Sep 23;14:172. doi: 10.1186/s13102-022-00563-6 (PMC9502946; doi:10.1186/s13102-022-00563-6)
Supplement: Supplementary file 2 — Additional File 2: Distribution of the four items on disordered eating behaviour. This pdf-file presents the distribution of the four items on disordered eating behaviour in numerical form. [file 13102_2022_563_MOESM2_ESM.pdf]

Additional file 2 regarding manuscript

Eating disorder risks and awareness among female elite cyclists: an anonymous survey

Distribution of the four items on disordered eating behaviour

1. Gone on eating binges where you feel that you may not be able to stop?*	Freq.	Percent	Cum.
never	39	32.23	32.23
once in a month	45	37.19	69.42
2-3 times a month	21	17.36	86.78
once a week	8	6.61	93.39
2-6 times a week	5	4.13	97.52
once a day and more	3	2.48	100.00
Total	121	100.00	

2. Ever made yourself sick (vomited) to control your weight or shape?	Freq.	Percent	Cum.
never	103	85.12	85.12
once in a month	5	4.13	89.26
2-3 times a month	9	7.44	96.69
once a week	2	1.65	98.35
2-6 times a week	1	0.83	99.17
once a day and more	1	0.83	100.00
Total	121	100.00	

3. Ever used laxatives, diet pills or diuretics to control your weight or shape?	Freq.	Percent	Cum.
never	109	90.08	90.08
once in a month	7	5.79	95.87
2-3 times a month	2	1.65	97.52
once a week	1	0.83	98.35
once a day and more	2	1.65	100.00
Total	121	100.00	

4. Exercised more than your coach wanted to lose or control your weight?	Freq.	Percent	Cum.
never	58	47.93	47.93
once in a month	22	18.18	66.12
2-3 times a month	22	18.18	84.30
once a week	12	9.92	94.21
2-6 times a week	6	4.96	99.17
once a day and more	1	0.83	100.00
Total	121	100.00	
